# ReGeneraTing Agents (RGTA ^®^) are a new option to improve amputation outcomes in the recovery of severe hand injuries

**DOI:** 10.1002/ccr3.1797

**Published:** 2018-09-14

**Authors:** Roohi Sharifah Ahmad, Denis Barritault

**Affiliations:** ^1^ Dept. of Orthopaedics Faculty of Medicine and Health Sciences University Putra Malaysia Serdang Malaysia; ^2^ Hand & Upper Limb Centre Pantai Hospital Kuala Lumpur Kuala Lumpur Malaysia; ^3^ OTR3 Paris France; ^4^ Laboratory CRRET UPEC 4397/ERL CNRS 9215 Université‐Paris‐Est‐Créteil Créteil France

**Keywords:** amputation of digits, CACIPLIQ, functional recovery, heparan sulfate mimetic, matrix therapy

## Abstract

CACIPLIQ20^®^ was used to accelerate the healing process and stimulate the viability of flaps and skin grafts, thereby improving amputation outcomes. An excellent range of motion was achieved with hardly any contracture or scarring. Pain relief and reduced sensitivity was noted, while healing of bone and tendon also improved, resulting in functional recovery.

## 1 INTRODUCTION

Six patients with distal digital amputations of Tamai I to IV and one with severe injury were treated with CACIPLIQ20^®^, a ReGeneraTing Agent (RGTA) that mimics heparan sulfates. Accelerated healing avoided severe outcomes allowing functional recovery. This is the first reported case series of hand injuries successfully treated with RGTAs.

In the current practice of hand surgery, we are often confronted with emergency cases presenting with significant trauma and soft tissue injury, or complications from initial treatment either at the local hospital or by the primary care physician that usually result in poor outcomes. In the case of revision amputations of the fingertip, loss of skin, pulp, fingernail, and digit length may all lead to functional deficits. A systematic review in 2013 of outcomes of fingertip revision amputations concluded that on average, fingertip revision amputation can achieve almost normal sensibility and satisfactory motion and patients can expect to return to work on average approximately 7 weeks after surgery. Thus, for patients who need to return to work within 2 months, fingertip revision amputation is recommended.^1^

Here, we had the opportunity to use a new product named CACIPLIQ20^®^, which is based on matrix therapy and recommended to treat chronic wounds in diabetic patients.^2^, ^3^ Matrix therapy using RGTA^®^ (ReGeneraTing Agent) is an innovative, minimally invasive approach in the field of regenerative medicine that aims to promote tissue regeneration by reconstructing the cellular microenvironment following tissue injuries.^4^ CACIPLIQ20^®^ contains OTR4120, a RGTA^®^ specifically formulated and dedicated for skin and plastic surgery.^5^, ^6^

Generally, vascularity of the upper extremity is much better when compared to that of the lower extremity, and ischemia is usually not an issue. However, in the case of amputation, ring avulsion, and full thickness skin loss (FTSL), skin and tissue coverage may pose a challenge, especially if the bed is avascular or infection is present. In the hand, wound healing by scarring with resulting contractures is a common but highly undesirable complication; hence, we are always fighting against time and scar tissue, looking for solutions to overcome this unfavorable outcome.

Here, we present a selection of seven cases with open wounds of full thickness dermal loss or dermal and subcutaneous loss of the upper limb, where conventional treatment methods had failed or no alternative acceptable treatment was available. Normally, these patients’ wounds would result in a poor outcome and require (more proximal) amputation. Initially, CACIPLIQ20^®^ was used as a last resort to avoid a dismal outcome. However, it was progressively used at earlier stages to improve outcome as well as functionality.

## 2 METHODS

### 2.1 Patients

All seven cases were chosen after a specific surgical index procedure was performed in the emergency setting where the wounds were either present primarily or developed secondarily due to failure of conventional treatment (debridement, antibiotics, and dressing) in the primary setting. CACIPLIQ20^®^ was applied in the described fashion to compromised patients (diabetic, chronic smokers) or ischemic wounds (amputations, full thickness loss) once the wounds were found to be nonhealing or regressing.

### 2.2 Materials

CACIPLIQ20^®^ is the commercial name for a RGTA^®^‐based skin healing agent. It is CE marked and commercially available in Malaysia. RGTA^®^s are heparan sulfate (HS) mimetics specifically designed to replace degraded heparan sulfates in damaged tissue, accelerating the speed and enhancing the quality of tissue repair.^4^ Their unique properties have been the subject of intensive preclinical and clinical studies.^2^, ^3^, ^6^, ^7^, ^8^, ^9^, ^10^, ^11^, ^12^ CACIPLIQ20^®^ contains RGTA^®^ OTR4120, a biodegradable polymer of 1‐6 alpha polyglucose substituted with *carboxymethyl* and sulfated groups.^4^ RGTA^®^ is biodegraded when internalized and catabolized through lysosomal pathways within cells, as with any other matrix element.

### 2.3 Intervention

Twice weekly application of a sterile gauze soaked with CACIPLIQ20^®^ was placed on the wound for 12 minutes and then removed.^1^ The wound was then covered with a nonocclusive dressing. When required, debridement, local antibiotics, and appropriate dressing materials were substituted or used in conjunction with before or after the intervention.^1^

## 3 RESULTS

### 3.1 Case reports: Amputations

A total of seven patients (two females and five males; between 26 and 68 years of age) were admitted to the hospital with hand injuries that led to a revision amputation. Application of CACIPLIQ20^®^ was initially started at POD 23(postoperative day), but progressively initiated earlier with the last patient beginning treatment on POD 1, for an average of 11.7 days. Three patients were smokers and two were diabetics (one well‐controlled and another newly diagnosed). The most common indication was ischemia with failure of FTSG (Full Thickness Skin Graft) uptake (5 of 7 patients). The clinical data are summarized in Tables 1 and 2 and described in detail in each figure. The level of amputation for each patient has been described according to the Tamai classification^13^ and included in Table 2.

**Table 1:** Summary of amputation cases

Case number	Age/gender	Comorbidity	Type of lesion	1st or 2nd intention and history	Infection Y/N	Ischemic Y/N	Start of CCPL (d)	Estimated success evaluation
1 NL	26/M	S	Ring avulsion amputation of RF at base of P1 with FTSG	1st, Ring FTSG	N	Y	POD23	Full take of “ring” FTSG allowing preservation of PIP joint
2 CWH	29/M	None	Grazed dorso‐radial IF with composite loss of bone, cartilage, ex‐ tensor tendon and capsule	1st, external fixators, FTSG and capsule repair	N	Y	POD17	Full healing, with no remnant pain in the PIP joint and reduced sensitivity at tip
3 KKM	51/M	DM	Crushed IF and MF	1st, external fixator, necrosis starting at both finger tips	N	Y and necrosis	POD14	Minimal improvement after revascularization and CCPL but amputation could not be avoided, earlier intervention may have avoided this AMP.
4 YPE	68/F	DM HT	MF tip amputation followed by flap necrosis	1st, flap and FTSG	N	N	POD9	Reversed fingertip necrosis
5 YT	48/M	S+	Thumb ring avulsion amputation and flap necrosis	2nd, FTSG, flap tip necrosis after 1 wk	N	Y	POD12	Revascularization and reversion of necrosis and full healing when CCPL was applied. Recovery of interphalangeal joint movement
6 LSC	46/M	Ex S+	Amputation of P3 of MF and RF with ischemic FTSG	1st, FTSG	N	N	POD6	Early application of CCPL reduced pain and enhanced good movement of the joint
7 LHY	32/F	None	Amputation of the tip of the RF Laceration of the SF	1st, FTSG	N	Y	POD1	Avoid necrosis of fingertip and accelerated skin graft

AB, antibiotic; AMP, amputation; CCPL, CACIPLIQ20^®^; DM, diabetes mellitus; FTSG, full thickness skin graft; HT, hypertension; IF, index finger; MF, middle finger; P1, proximal phalanx; P2, middle phalanx; P3, distal phalanx; POD, postoperative day; RF, ring finger; S, medium smoker; S+, heavy smoker; SF, small finger.

**Table 2:** Patient characteristics

Case number	1 NL	2 CWH	3 KKM	4 YPE	5 YT	6 LSC	7 LHY
Tamai classification	III	IV	III	I	II	II	III
Evaluation of blood supply	Ischemic	Ischemic	Ischemic	Ischemic	Ischemic	Ischemic	Ischemic
TAM	175	150	190	210	120/150	190	190
TAM % (out of 260 degrees)	67.5%	57.7%	73.1%	80.8%	80%	73.1%	73.1%
TAM % (out of 190 degrees)	92.1%	79%	100%	80.8%	80%	100%	100%
Blood sugar	NA	NA	Newly diagnosed	Well controlled	NA	NA	NA
Patient satisfaction score	7	8	7	8	9	8	6

## 4 DISCUSSION

### 4.1 By patient

**Figure 1:**
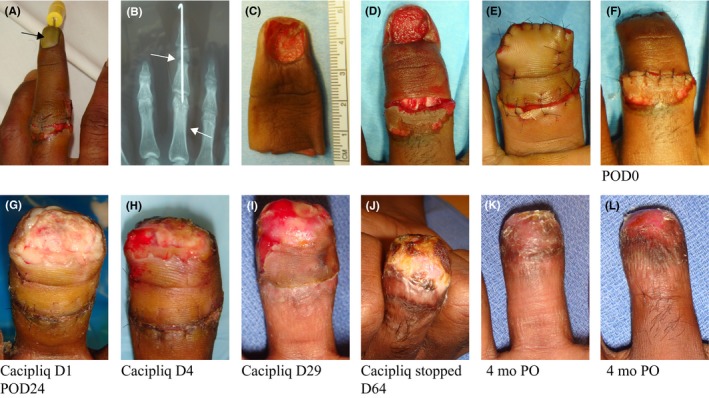
Patient 1: Ring avulsion with FTSG. A, A 26‐year‐old lorry driver presented 20 h postring avulsion for amputation of his ^®^ MF after it had been wired and simply sutured elsewhere! The tip was already dusky. B, The skin and soft tissue envelope were avulsed from the proximal phalanx; however, there was a fracture of the neck of P2 (Tamai III). We decided to try to salvage the PIP joint, but it needed cover. C, A Full thickness “ring graft” was scavenged from the amputated part and applied to D, the remaining stump, which was ischemic distally. E, F, Immediate post‐op. G, 3 wk later, (the entire stump was white) CACIPLIQ20^®^ was started. H, Within 3 d, neovascularization was seen. I, In 3 wk, the entire 1.2 cm^2^ of stump was covered with granulation tissue. J, CACIPLIQ20^®^ was stopped at 2 mo, and the dorsal skin was covered and supple, allowing flexion at the PIP joint. K, L, 4 mo post‐op and after coban stump molding, the patient was ready for prosthesis fitting. FTSG, full thickness skin graft; MF, middle finger; PIP, proximal interphalangeal

Patient 1 presented with an amputated digit of more than 20 hours of warm ischemic time. While revision amputation at proximal phalanx level (P1) would have been the recommended treatment at this stage, we decided to attempt to salvage the PIP joint since the middle phalanx (P2) was still vascularized. At 3 weeks, there were no signs of acceptance of the full thickness “ring” skin graft, partly because the patient was a smoker (which he only revealed later). Treatment with CACIPLIQ20^®^ was the last resort before a more proximal amputation would be required. A single application was sufficient to initiate neovascularization of the ischemic tissue and the appearance of granulation tissue. Thus, the intervention definitely contributed to the preservation of the PIP joint and 20 mm of digital length, which greatly enhanced functional possibilities (flexion of the PIP joint). Another point to note is that healing by granulation usually results in poor tissue quality and susceptibility to breakdown, especially over the joints, as well as contracture, resulting in reduced mobility. Here, although dorsal healing was by secondary intention and granulation tissue, the coverage appeared to be strong, supple, and mobile over the PIP joint and certainly attributable to the regenerative capacity of the applied product (Figure 1).

**Figure 2:**
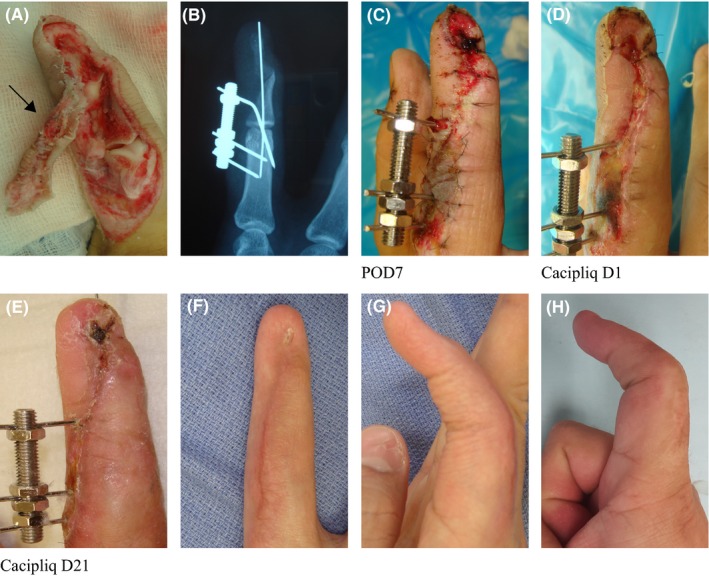
Patient 2: Grinding injury of Index Finger (IF) A, A 29‐year‐old right‐handed mechanical engineer caught his IF in a fast‐moving roller band. The dorso‐radial half was shaved off like a friction burn, exposing and destabilizing both the DIP and PIP joints (Tamai IV) due to loss of the radial half of the P2 and dorsal half of P3. Only a piece of frayed skin (arrow) was attached; thus, consent for amputation was taken. B, The joints were skeletally stabilized with a fine K‐wire and external fixator. C, The dorsal capsules were repaired tight to simulate the extensor mechanism and covered with the (frayed) skin graft. D, Upon seeing infection and discoloration, the dead skin was removed, wound debrided, and CACIPLIQ20^®^ was applied (POD17) to hasten closure. E, Within three (3) weeks, the wound healed. F, At 6 weeks, the external fixator was removed and therapy started. G, At 15 weeks PT (posttrauma), there was 45° of flexion at the PIP joint. H, After bony augmentation and fusion of the DIP joint, he regained 10‐75° of pain‐free PIPJ motion. At 4 years follow‐up, he had 4 kgF pinch and 4 mm 2P.D. DIP, distal interphalangeal; IF, index finger; PIP, proximal interphalangeal; PIPJ, proximal interphalangeal joint; POD, postoperative day

Patient 2 had an amputation which could not be replanted due to a severe grinding injury claiming the radial half of his dominant index finger (IF). Thus, every effort had to be made to restore form and function as much as possible. Use of the regenerative agent was therefore crucial for several reasons: first, for salvaging the skin graft and wound closure; second, for reducing the hypersensitivity of the digit; and third, for helping to maintain/regain the shape and form of an IF. Since the radial digital nerve had been sheared off (Figure 2), the return of 2‐point discrimination to 4 mm—although not scientifically provable—also seems likely to be due to the effects of the regenerating agent.

**Figure 3:**
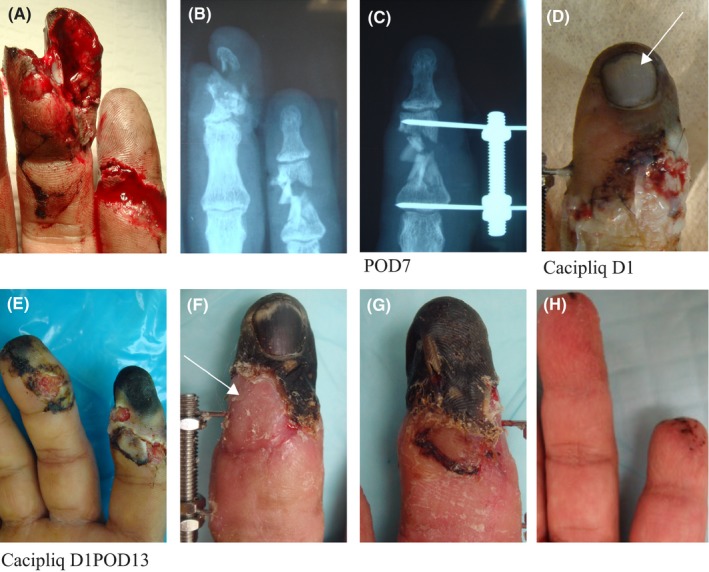
Patient 3: Mechanically crushed ischemic digit. A, B, A 51‐year‐old male had his dominant hand cut by a chainsaw‐like machine, severing both digital arteries of the IF (Tamai III) and crushing the MF tip. C, The radial digital artery was repaired just distal to the PIPJ and the P2 bridged with a LINK ^®^ fixator. D, Four days later, some ischemia was seen at the tip, more on the ulnar side. E, Delayed start of CACIPLIQ20^®^ at POD 13. F, G, At 6 weeks, the minimal gain in vascularity was enough so that a radial flap (arrow) could be fashioned to cover H, the revision amputation. DIP, distal interphalangeal; IF, index finger; MF, middle finger; PIPJ, proximal interphalangeal joint; POD, postoperative day

Patient 3 had a severe ischemic insult with a crush element to his right index and middle fingers. Initial surgical repair (MF) and revascularization (IF) achieved some restoration of the blood supply (Figure 3D). However, these interventions failed to prevent tip necrosis, possibly due to diabetic vasculopathy. CACIPLIQ20^®^ was applied 2 weeks later in hopes of halting necrosis and restoring further blood supply to the index finger. Although the minimal improvement could not prevent amputation of the tip of the IF P3 (Figure 3F,G), it enabled the radial flap to be fashioned to provide cover (Figure 3H). Perhaps, an earlier intervention, when the ischemia first manifested (Figure 3D), would have resulted in a better outcome (Figure 3).

**Figure 4:**
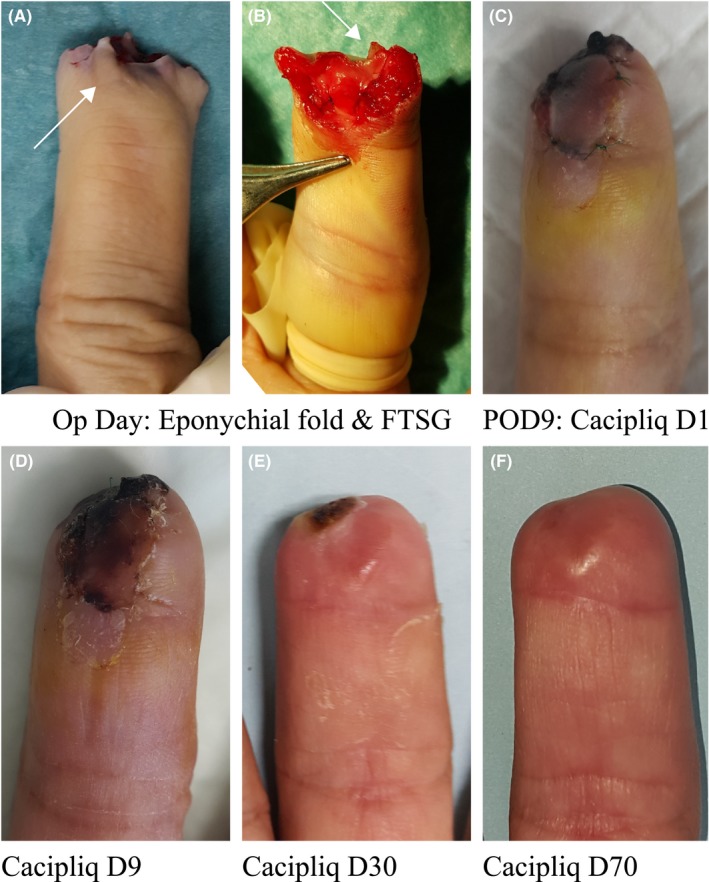
Patient 4: Fingertip amputation with flap necrosis. A, Amputation following right MF caught in a door jamb in a 68‐year‐old diabetic patient just distal to the nail fold (Tamai I, arrow). B, A revision amputation was done with minimal shortening and an eponychial flap designed for cover (arrow) along with a full thickness graft from the amputated part. C, This looked dusky 9 d later (POD9), and CACIPLIQ20^®^ was started. D, Within 9 d, the color changed and union was seen. E, Excellent healing within a month. F, A smooth nonsensitive functional fingertip. MF, middle finger; POD, postoperative day

Patient 4's amputated finger healed well considering a distal flap was used as well as a skin graft in a diabetic patient. This could be attributable to the early application of the regenerative agent, although the patient had reasonably good blood sugar control. Therefore, we advocate early application in order to achieve optimal benefits (Figure 4).

**Figure 5:**
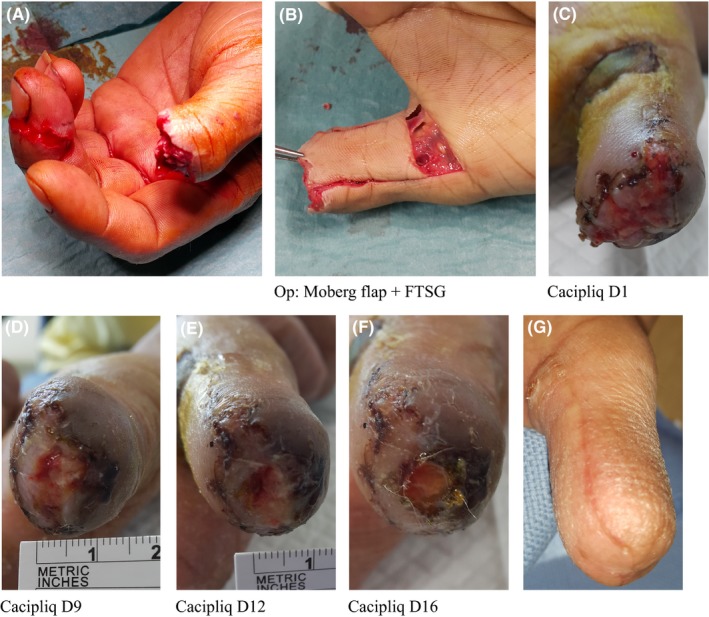
Patient 5: Ring avulsion amputation with ischemic flap. A, A 48‐year‐old foreman—heavy smoker of 60 pack years—sustained a jagged avulsion amputation of his right dominant thumb (Tamai II) by a ribbed coil while installing a manhole cover. B, To cover the revision amputation, a Moberg flap was designed and the base was elevated, placing a FTSG taken from the amputated part. C, A week later, the FTSG was doing well; however, there was partial necrosis of the closure at the tip, as expected due to his smoking habit. CACIPLIQ20^®^ was started at POD 12 on a deficit measuring 10 by 15 mm. D, Within 9 d, the color was much better and epithelial ingrowth could be seen. E, The size was halved by D12 and again halved by day 16. Closure achieved by D33. F, Well molded at 2.5 mo with an IPJ ROM of 0‐45° and a 2P.D. of 4‐5 mm at the tip. No hypersensitivity present. FTSG, full thickness skin graft; POD, postoperative day

Patient 5 The regenerative agent was applied on the 12th day, and closure was achieved in a flap in 30 days for this patient, who is a heavy smoker. This excellent cosmetic and functional result (well‐padded and molded tip without sensitivity) was achieved in a comparatively short period, especially when considering all the negative factors. The second benefit was the maintenance of the flap quality—the skin and tissues did not thin out as what normally occurs during the healing of ischemic areas. The final benefit was the normal 2‐point discrimination (Figure 5).

**Figure 6:**
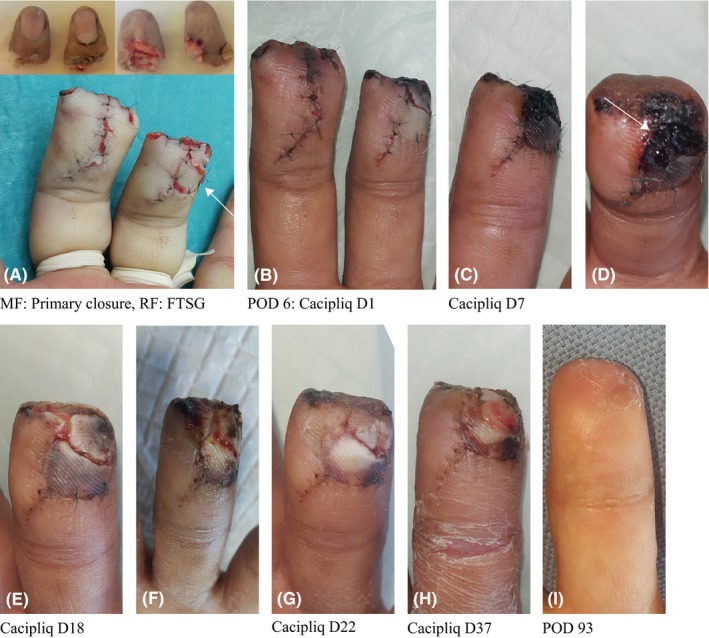
Patient 6: Double fingertip amputation at DIPJ level with ischemic FTSG. A, In a 46‐year‐old smoker with a heavy machine press (crush) injury, revision amputation is the treatment of choice. The MF could be closed primarily, and the RF needed a FTSG (arrow) which was salvaged from the amputated digit. B, At POD 6, the RF graft was pale, and CACIPLIQ20^®^ was started. C, A dusky graft was due to an underlying hematoma lifting off the FTSG. D. View of the underlying hematoma E, The stitches were removed, wound washed, and Steri‐Strip used for closure. F, A week later, the flap is pale and weepy but the FTSG has taken proximally. G, Finally, flap and FTSG taken. H, 5 wk of CACIPLIQ20^®^. I, 3 mo post‐op. FTSG, full thickness skin graft; MF, middle finger; POD, postoperative day; RF, ring finger

Patient 6. The regenerative agent was applied relatively early on the 6th postoperative day, but it still took 37 days in total to achieve closure in an ex‐smoker. The tips were well‐padded with no sensitization, and there was good movement in the PIP joints. In this patient, although healing was good, it was not as rapid as expected with the use of CACIPLIQ20^®^ (Figure 6).

**Figure 7:**
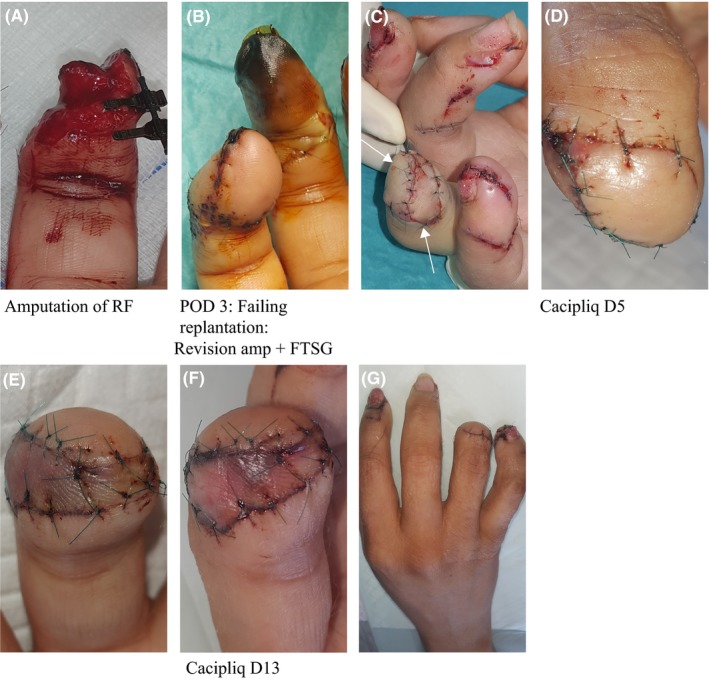
Patient 7: Revision amputation, flap, and FTSG. A, A 32‐year‐old manager caught her ^®^ dominant hand in an industrial fan injuring all four fingers with an amputation of her RF. B, Replantation failed after 30 h. C, However, a 1‐cm gain in length was achieved because a dorsal flap (top arrow) survived. A day after the surgery, CACIPLIQ20^®^ was started mainly for the FTSG (bottom arrow). D, E, At 5 d, the flap was pink, but the FTSG still a bit dusky. A second application ensured a good outcome. F, The FTSG and the flap are both pink. G, All the fingers after 2 wk. FTSG, full thickness skin graft; RF, ring finger

Patient 7 was in a very emotional state and upset about the amputation. Since the skin was taken from the necrosing fingertip, CACIPLIQ20^®^ was applied immediately after the revision amputation to ensure the uptake of the skin graft and a good outcome (Figure 7).

### 4.2 Overall

The advantages and disadvantages of RGTA usage are summarized in Table 3. The most dramatic effect documented was in the first patient where new vessels formed (neovascularization) after the application of the agent in completely nonvascularized tissue, even 23 days after the primary event occurred.

**Table 3:** Advantages and disadvantages of Cacipliq usage

Advantages	Neovascularization develops in ischemic tissue Regeneration of like‐for‐like skin reduces stiffness/contractures Accelerated healing, saves cost in long‐term Helps preserve length of digit—salvage of dying skin graft, flap Reduces hypersensitivity in fingertip amputations Returns 2 P.D to normal in certain injuries Enhances bone and tendon healing Helps to maintain/regain shape
Disadvantages	Contraindicated for people with cutaneous type 4 hypersensitivty to heparin and heparinoids If wound healing occurs too quickly, latent osteitis remains undetected and can eventually lead to outbreak of infection. Can be potentially costly if spray format is unavailable (availability is country‐dependent)

In addition, the skin quality that developed was very much native to the defect and had excellent mobility over the joints, allowing near‐complete range of motion (where three patients had 100%, one 92.1%, and the rest (three) approximately 80%) with hardly any contracture or scarring seen.

Finally, rapid healing of the wounds was observed across the board and with some cases (within the same patient) over time. Rapid healing was seen in nondiabetics and nonsmokers but diabetic patients also healed faster than expected. Smokers had a delay in the healing process (30 days or more for fingertip injuries), but this would either not have healed or taken much longer otherwise. Return of circulation to dusky flaps (1) and full thickness skin grafts (5) was clearly documented and enabled preservation of length in digits as well as joint function.

Pain was addressed in the sense that dressing time and duration were reduced; however, a pain score, though reduced with this intervention, was not recorded in all patients. It was noted that the hypersensitivity that usually occurs in fingertip injuries was absent. Return of 2‐point discrimination to normal (4‐5 mm) in patients 2 and 5 was an unexpected bonus. The digital nerve was sheared off in the former, and the revision amputation was just distal to the IP joint in the latter. The only plausible explanation is some regeneration of nerve fibers.

Bone healing and tendon healing were also improved as illustrated by motion recovery (Figure 2G,H). This is in accordance to preclinical studies on nerve, bone, and tendons^3^, ^13^, ^14^21 and with some nonpublished clinical observations.

The only point to be aware of is that debridement is absolutely a prerequisite. Although the wounds with mild infection or slough did heal, healing was faster when infection or slough was not present. Thus, an infection first has to be cleared with either local or systemic antibiotics before healing can take place. Also, bacterial biofilm may grow and if not removed efficiently through the debridement procedure will prevent access of CACIPLIQ20^®^ to the damaged matrix, thus inhibiting its treatment potential. In certain cases, approximation of tissue was aided by supportive tapes to enhance the process. Various dressings may need to be used as an adjunct to deal with the different types of wounds.

## 5 CONCLUSION

RGTA^®^ therapy induced significant wound healing in patients with either severe ischemia or no blood supply. It also sped up the healing process in smokers and diabetics. The bonus points were little or no scarring, which allowed for increased mobility over joints and maintenance of suppleness. Further studies need to be done to assess the effect on nerve and tendon healing.

## CONFLICT OF INTEREST

DB has financial interest as inventor of patented RGTA^®^ technology but other co‐authors have none.

## AUTHOR CONTRIBUTION

SAR: involved in the conception and design of the study, acquisition of data, analysis and interpretation of data. RSA and DB: drafted and finalized the article.

## ETHICS AND RESEARCH APPROVAL

The patients provided written informed consent as per the Hospital's institutional review board guidelines. As CACIPLIQ20^®^ is available on the market as a healing agent and only individual cases are reported as such, no specific authorization was requested.

## Endnote

1 The CACIPLIQ20^®^ used in the study was a liquid form sterile‐packed in a 5‐cc bottle. At present, it is worthwhile to note that a more user‐friendly sterile form (CACIPLIQ^®^SPRAY) has been made available which can just be sprayed onto the wound.
